# Role of Human Leukocyte Antigen System as A Predictive Biomarker for Checkpoint-Based Immunotherapy in Cancer Patients

**DOI:** 10.3390/ijms21197295

**Published:** 2020-10-02

**Authors:** Francesco Sabbatino, Luigi Liguori, Giovanna Polcaro, Ilaria Salvato, Gaetano Caramori, Francesco A. Salzano, Vincenzo Casolaro, Cristiana Stellato, Jessica Dal Col, Stefano Pepe

**Affiliations:** 1 Department of Medicine, Surgery and Dentistry ’Scuola Medica Salernitana’, University of Salerno, 84081 Baronissi, Salerno, Italy; fsabbatino@unisa.it (F.S.); gpolcaro@unisa.it (G.P.); il.salvato22@gmail.com (I.S.); frsalzano@unisa.it (F.A.S.); vcasolaro@unisa.it (V.C.); cstellato@unisa.it (C.S.); spepe@unisa.it (S.P.); 2 Oncology Unit, AOU San Giovanni di Dio e Ruggi D’Aragona, 84131 Salerno, Italy; 3 Department of Clinical Medicine and Surgery, University of Naples “Federico II”, 80131 Naples, Italy; luigiliguori1992@gmail.com; 4 Pulmonary Unit, Department of Biomedical Sciences, Dentistry, Morphological and Functional Imaging (BIOMORF), University of Messina, 98125 Messina, Italy; gaetano.caramori@unime.it

**Keywords:** major histocompatibility complex (MHC), human leukocyte antigen (HLA), antigen processing machinery (APM) molecules, carcinogenesis, tumor predisposition, biomarker, cancer immunotherapy

## Abstract

Recent advances in cancer immunotherapy have clearly shown that checkpoint-based immunotherapy is effective in a small subgroup of cancer patients. However, no effective predictive biomarker has been identified so far. The major histocompatibility complex, better known in humans as human leukocyte antigen (HLA), is a very polymorphic gene complex consisting of more than 200 genes. It has a crucial role in activating an appropriate host immune response against pathogens and tumor cells by discriminating self and non-self peptides. Several lines of evidence have shown that down-regulation of expression of HLA class I antigen derived peptide complexes by cancer cells is a mechanism of tumor immune escape and is often associated to poor prognosis in cancer patients. In addition, it has also been shown that HLA class I and II antigen expression, as well as defects in the antigen processing machinery complex, may predict tumor responses in cancer immunotherapy. Nevertheless, the role of HLA in predicting tumor responses to checkpoint-based immunotherapy is still debated. In this review, firstly, we will describe the structure and function of the HLA system. Secondly, we will summarize the HLA defects and their clinical significance in cancer patients. Thirdly, we will review the potential role of the HLA as a predictive biomarker for checkpoint-based immunotherapy in cancer patients. Lastly, we will discuss the potential strategies that may restore HLA function to implement novel therapeutic strategies in cancer patients.

## 1. Human Leucocyte Antigen and Antigen Presentation Machinery Molecules: An Overview

### 1.1. HLA Class I: Structure and Function

The major histocompatibility complex (MHC), better known in humans as human leukocyte antigen (HLA), is a very polymorphic gene complex encoding for cell surface molecules specialized to present and recognize self and non-self peptides [1,2,3,4,5,6,7,8]. HLA complex contains more than 200 identified loci located close together on a 3 Mbp stretch within the short arm of chromosome 6 [6,8,9]. Population surveys have identified several thousands of allelic variants of HLA molecules which mainly affect the nature and composition of their peptide-binding groove, regulating the peptide repertoire presented on the cell membrane [6,8,9,10]. These allelic variants can be associated with an increased risk of various diseases including cancer [11]. 

HLA is categorized into three groups on the basis of function and structure: class I, II and III [12,13]. HLA class I molecules are expressed on the surface of nucleated cells, except for germ line and some neuronal cells [14]. HLA class I molecules display on cell membrane peptide fragments derived from endogenously degraded self or non-self proteins to T-cell receptor (TCR) of CD8+ cytotoxic T lymphocytes (CTLs) [7,15,16,17,18]. Peptides derived from unmutated (self) proteins are normally ignored by CTLs, whereas those derived from mutated (self) or pathogen (non-self) proteins are recognized and trigger an adaptive immune response [15,16,17]. Particularly, tumor cells are characterized by mutated genes and aberrantly expressed cellular proteins from which derived tumor specific antigens (TSAs) and tumor-associated antigens (TAAs). Through the presentation of TAAs/TSAs, tumor cells become susceptible to CTL-mediated lysis. Using this immune surveillance system, CTLs eradicate intracellular pathogens and exert potent antitumor activity, eliminating the transformed or infected cells through the adaptive immune response [19]. In addition, HLA class I molecules can also present peptides generated from exogenous proteins, a process known as cross-presentation [14,20]. This process is necessary to recognize and destroy tumor cells as well as viruses that do not readily infect antigen-presenting cells, stimulating naïve T cells into activated CTLs [14,20,21]. Specifically, cross-presentation involves dendritic cells (DCs) which present TAAs/TSAs or pathogen-derived peptides in their HLA class I complex to naïve T cells [22,23,24]. Extracellular peptide loading on HLA class I complex differs from the canonical way followed by intracellular peptides and it will be discussed below in this review. However, recently, several lines of evidence demonstrated that macrophages are also able to implement a cross-presentation process, subverting the original belief that it is an exclusive characteristic of DCs [22].

Besides mediating an adaptive immune response, HLA class I molecules also play a key role in the innate immune response since they serve as ligands of inhibitory killer cell immunoglobulin-like receptors (KIRs) of Natural Killer (NK) cells [25]. Because the majority of healthy nucleated cells express HLA class I molecules, inhibitory KIRs ensure that NK cells do not attack normal cells which express HLA class I molecules but eliminate infected and tumor cells which may have reduced expression of HLA class I molecules [25,26]. Structurally, HLA class I molecules are heterodimers that consist of two polypeptide chains, alpha (α) heavy chain and β2-microglobulin (β2-m) light chain [27]. The β2-m subunit is not polymorphic and is encoded on human chromosome 15. In contrast, the α chain is polymorphic and is encoded by HLA class I genes, further categorized in HLA-A, -B, and –C, according to the locus of their encoding gene [28,29,30]. The α heavy chain has three extracellular domains (α 1-3, with α1 being at the N-terminus), a transmembrane region and a C-terminal cytoplasmic tail [30,31,32]. The only invariant region is the Ig-like α3 domain, essential for non-covalent association with the β2-m light chain [30,31,32]. The α3-CD8 interaction holds the HLA class I molecules in place, while the TCR binds to α1-α2 and checks the coupled peptide for antigenicity [30,32]. The α1 and α2 domains fold to make up a groove for peptides to bind. Bound peptides are predominantly 8-10 amino acid in length, but longer peptides have also been reported [30,32,33].

Besides HLA-A, B, and C, some other HLA class I molecules are also encoded by non-classical HLA loci. Those include HLA-E, which primarily presents various peptides that are derived from the leader sequence of some HLA class I molecules. It blocks conventional NKs expressing the inhibitory heterodimeric NKG2A/CD94 receptor. Lastly, HLA-F mainly resides intracellularly and rarely reaches the cell surface; HLA-G, plays a role in protecting the fetus from the maternal immune responses [34,35,36,37,38,39].

### 1.2. HLA Class I Antigen Processing Machinery Complex and Antigen Presentation

The generation and expression of HLA class I antigen-derived peptide complexes is a multistep process and requires an integral and functional HLA class I antigen processing machinery (APM) [25]. This is constituted by several distinct components, such as the proteasome complex, the ubiquitination system, the transporters associated with antigen processing (TAP)1 and TAP2, the endoplasmic chaperone molecules (calnexin, calreticulin, ERp57, and tapasin), and the Golgi apparatus [25,40,41,42]. The generation and expression of HLA class I antigen derived peptide complexes and their presentation to naïve CD8+ T cells require four main tasks: (i) peptide generation and trimming; (ii) peptide transport; (iii) assembly of the HLA class I loading complex; and (iv) antigen presentation [25,30,43,44,45,46,47,48]. Firstly, proteins are targeted for degradation by the covalent attachment of multiple copies of the 76-residue protein ubiquitin to free amino groups of Lys [25,49]. Subsequently, they are transferred to the proteasome, where the catalytic core, called the 20S proteasome, contains α and β subunits. The catalytic core interacts with regulatory particles and creates a physical barrier to regulate access to the gate. The latter has protease catalytic activity [25,50,51,52]. Three of the 20S proteasome’s β subunits δ(β1), Z(β5), and MB1(β2) may be replaced by the functionally different counterparts low molecular proteins (LMP) as LMP2 (also called β1i), LMP7 (β5i), and LMP10 (β2i), respectively [25,53,54,55]. Proteasome incorporating LMP2, LMP7 and LMP10 is called immunoproteasomes because it develops under conditions of intensified immune response [25]. The immunoproteasome formation is induced during inflammation by stimulation with type I (α and β) or type II (γ) interferons (IFNs) [25,56,57]. Moreover, the immunoproteasome is involved in other activities such as generation of cytokines as well as regulation of T cell differentiation, survival and function during thymocyte development [25,58,59]. Peptides generated in the proteasome are then actively transported from the cytosol into the endoplasmic reticulum (ER) lumen by TAP [25]. TAP is a heterodimeric complex composed of two half-transporters, TAP1 and TAP2, members of the adenosine triphosphate (ATP)-binding cassette transporter family. This complex forms a transmembrane pore in the ER membrane whose opening and closing depend on ATP binding and hydrolysis, respectively (ATP switch model) [25,60,61,62,63]. TAP transports most efficiently peptides of a well-defined length (8–12 residues), while longer peptides can be further trimmed in the ER lumen or, alternatively, can be transported back to the cytosol where they are trimmed by cytosolic peptidases and recycle back to the ER [25,64,65,66,67,68,69,70]. Peptides transported into the ER by TAP are loaded onto nascent HLA class I molecules with the assistance of four chaperone proteins: calnexin, the thiol oxidoreductase ERp57, calreticulin, and tapasin [25,71,72,73,74,75,76,77,78,79]. Specifically, the HLA class I α heavy chain interacts with calnexin, which facilitates its complete folding and, by acting in concert with ERp57, ensures the correct oxidation [25,80,81]. At this point, the conformation of the α heavy chain is recognizable by β2-m [25,82]. Their binding triggers the release of calnexin [25,82,83]. The resulting conformational changes give the α heavy chain/β2-m heterodimer an “open” form that interacts with calreticulin [25,74]. High affinity peptide binding requires the additional participation of tapasin, which links the complex to nascent HLA class I molecules [25,75,79]. After peptide loading, HLA class I derived peptide complex dissociates from TAP as well as from ER-resident chaperones and clusters at export sites on the ER membrane, where it is selectively recruited into cargo vesicles for transport to the Golgi apparatus and then to the cell membrane [25]. On the membrane, the HLA class I derived peptide complex is extracellularly exposed to be recognized by the TCR of naïve T cells, potentially triggering an adaptive immune response when non self or mutated self antigen derived peptides are expressed [19,25].

During cross-presentation extracellular antigens need to enter into canonical HLA class I route and they can exploit various ways. (i) Extracellular peptides can be directly transferred from infected or tumor cells to the cytosol of DCs through the Gap junctions. (ii) ER components can fuse with endosomal/phagosomal pathway and the exogenous peptides are exported from phagosome into cytosol through the ER-associated protein degradation system. (iii) Recycling HLA class I molecules are loaded with extracellular peptides into recycling endosome. (iv) Exosomes secreted by infected or tumor cells can directly bind to DCs [84,85]. 

### 1.3. HLA Class II: Structure and Functions

In contrast to HLA class I molecules, HLA class II molecules are usually present only on professional antigen-presenting cells (APCs) (B cells, macrophages, DCs, Langerhans cells), thymic epithelium and activated (but not resting) T cells [86,87,88]. In all other nucleated cells, HLA class II antigen expression can be induced by IFN-γ [88,89,90]. HLA class II molecules promote the switch of naïve T cells into activated T cells by presenting exogenously derived antigen peptides to CD4+ T cells [86,87,88,89,90]. Moreover, HLA class II molecules regulate the functions of B cells, macrophages and T cells [87,88,91]. They are encoded by genes in the HLA-DP, -DQ and -DR loci of the chromosome 6 cluster [6]. HLA class II molecules consist of 2 highly polymorphic polypeptides, the α and β chains [87,92,93,94]. Only the β2 domain of the β chain is a non-polymorphic region. It constitutes the binding site for the CD4+ T cell co-receptor [87,92,93,94,95,96]. HLA class II molecules have a peptide-binding domain, an Ig-like domain and a transmembrane region with a cytoplasmic tail and are responsible for binding peptides (15-24 amino acids) derived from extracellular sources [87,92,93,94,95,96]. Therefore, HLA class II binds peptides longer than HLA class I and accommodates peptide side chains within its binding pocket. These two features increase HLA class II peptide diversity [97,98].

### 1.4. HLA Class II Antigen Presentation System: How it Works

Compared to HLA class I, also the HLA class II antigen presentation process is characterized by different tasks. This involves several molecules and protein complexes [99,100]. Firstly, α and β chains are assembled in the ER with the invariant chain (li, CD74), forming the (α/β-li)_3_ complexes [99,100,101]. Invariant chain li occupies the peptide binding groove of HLA class II, preventing peptide loading within ER [98,102]. Li targets HLA class II containing vesicles to acidic endosomes. Then, into these acidic endosomes, called MHC class II compartments (MIICs), the li chain undergoes selective proteolytic digestion, forming the class II-associated I chain peptide (CLIP). This peptide occupies the groove of HLA class II dimers [100,103]. Subsequently, CLIP is exchanged by tightly bound peptides derived from proteins degraded into the endosomal pathway [99,100]. HLA-DM molecules are crucial to facilitate this exchange by promoting CLIP removal and stabilizing the peptide free status of HLA class II molecules. Moreover, HLA-DM also catalyzes the release of weakly bound peptide, ensuring that only strong bound peptide HLA class II complexes reach the cell surface [98]. Finally, the HLA class II derived peptides complexes are exposed on APCs [100,103,104]. 

### 1.5. HLA Class I and II Transcription Regulation

The transcription of genes encoding for the components of HLA class I and II complex is tightly regulated according with the crucial role of these molecules to obtain an effective adaptive immune response. HLA class I genes, except for HLA-G, contain several conserved *cis*-acting regulatory elements. Specifically, three different elements are important for both constitutive and inducible expression. The first element, called enhancer A, contains a binding-site for the nuclear factor kB (NF-kB). The second one corresponds to an IFN-sensitive response element (ISRE) and allows the binding of IFN Regulatory Factors 1 (IRF1). Lastly, the third one is an SXY module comprising four different boxes: W/S, X1, X2 and Y. Equally, the promoter of β2-m, but not those of other genes involved in antigen processing and presentation such as TAP or LMP, contains all three *cis*-acting regulatory elements in its proximal region [105]. Conversely, HLA class II gene proximal promoters contain only the SXY module which is bound in its X1 box by the regulatory factor X (RFX) complex, which comprises RFX5, RFX-associated ankyrin-containing protein (RFXANK) and RFX-associated protein (RFXAP). The cAMP-responsive element binding protein 1 (CREB1) and the activating transcription factor 1 (ATF1) bind the X2 box; the nuclear transcription factor Y (NFY) complex interacts with the Y box. Instead, the elements interacting with W/S box remains poorly defined [106,107]. The interactors with SXY module of genes belonging to both HLA class I and II are crucial elements in the transcriptional control. Indeed, since the identification in 1993 of the class II trans-activator CIITA [108] and, more recently of the NOD-like receptor 5 (NLRC5) [109], also called class I trans-activator CITA, it has been clearly highlighted that both these NLR proteins miss of a DNA-binding domain. Therefore, both NLRC5/CITA and CIITA need to cooperate with the multiprotein complex that is assembled on the SXY module to exert their transactivation activity (forming CITA- and CIITA enhanceosomes) [110,111]. Different studies exploiting CIITA-deficient mice [112] and several molecular analyses performed in patients affected by bare lymphocyte disease (BLS) with HLA class II deficiency confirmed CIITA as the master regulator of HLA class II expression [92,113,114]. Differently NLRC5/CITA is defined as a key regulator of HLA class I, especially in selected immune cell subsets. Indeed, the generation of NLRC5/CITA knockout mice in three independent studies has allowed to show a retention of HLA class I expression in professional APCs also in the absence of the trans-activator [115,116,117], suggesting the presence of a compensatory mechanism. These results agreed with previous findings regarding the ability of CIITA to contribute to HLA class I expression control [118]. Moreover, NLRC5/CITA regulates the expression of other genes involved in HLA class I presentation and processing, such as β2-m, LMP2, and TAP1 [109]. Interestingly, the up-regulation of both NLRC5/CITA and CIITA is critical for the efficient induction of HLA class I and II, respectively, by IFN-γ stimulation. The induction of NLRC5/CITA by IFN-γ precedes HLA class I gene expression as well as CIITA transcript levels are induced earlier than HLA class II genes upon IFN-γ stimulation [119]. 

### 1.6. HLA Class III: A Poorly Characterized Class

The structure and function of HLA class III molecules are poorly defined. They are not involved in antigen binding but in inflammatory processes. Their gene cluster is present between those of class I and class II molecules and encodes important molecules involved in inflammatory processes including complement components C2 and C4, factor B, tumor necrosis factor (TNF)-α, lymphotoxin, and heat shock proteins [12,120,121,122].

### 1.7. Carcinoma Cells as Non-Professional APC: A Novel Role for HLA Class II Complex

As mentioned above, HLA class II is usually express only on APCs’ surface, playing a crucial role in CD4+ T cell activation [86,87,88]. However, several lines of evidence showed that many type of cancer cells can also express MHC class II complex regardless tissue origin [123,124,125,126,127,128,129,130]. So far, the role of tumor specific MHC class II (tsMHC-II) expression remains unclear. Conflicting evidences are reported about how tsMHC-II regulates cancer progression as well as immune checkpoint inhibitor (ICI)-based immunotherapy response [98]. The expression of tsMHC-II has been related to longer progression free survival (PFS) and overall survival (OS) in melanoma and Hodgkin lymphoma patients treated with programmed death cell 1 (PD-1)/programmed death-ligand 1 (PD-L1) monoclonal antibodies (mAbs) [123,125,126]. In contrast, a similar association was not found in melanoma patients treated with a cytotoxic T-lymphocyte-associated protein 4 (CTLA-4) mAb [126]. Two independent studies on breast cancer specimens, evaluating tsMHC-II expression by both immunohistochemistry (IHC) and RNA sequencing demonstrated that tsMHC-II expression positivity correlated with longer disease free survival (DFS) and PFS [124,131]. These results were observed also in advanced-stage serous ovarian cancer [132]. However, further clinical trials are needed to define the role of tsMHC-II expression as a potential biomarker for ICI-based immunotherapy in cancer patients.

## 2. Defects and Clinical Significance of HLA in Human Cancer

### 2.1. HLA Class I Molecule Defects

**Figure 1:**
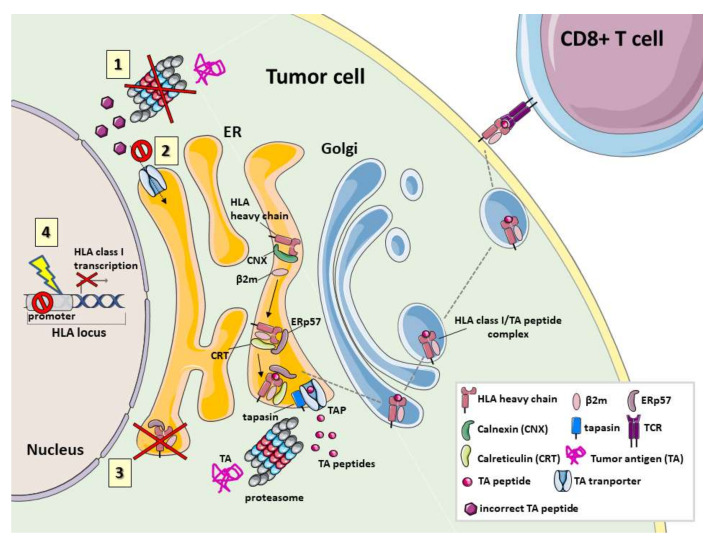
Defects in tumor antigen processing, translocation and loading on HLA class I. Normally tumor antigens (TAs) are degraded by proteasome/immunoproteasome into TA peptides and translocated into the endoplasmic reticulum (ER) through ATP-dependent activation of TAP transporters. Then, different chaperones: tapasin, calnexin (CNX), calreticulin (CRT) and ERp57 form a multimeric complex that provides for the correct assembly of HLA class I and for peptide loading. The lack of TA presentation during tumor development can be determined by different defective mechanisms depicted in the cartoon. **1**. Mutations in genes coding for proteasome subunits or deregulation of their expression implicate an incorrect TA degradation and the production of modified TA peptides. **2**. Mutations in TAP genes, associated with down-regulation of their expression or to their dysfunction, reduce the translocation of TA peptides into the ER. **3.** Defects in the expression of chaperones reduce the stable assembly of the “peptide-free” HLA class I molecule and of the HLA class I molecule-TA peptide complexes inhibiting a correct and efficient TA peptide presentation. **4**. Defects in HLA class I gene expression involve the total loss of these genes or mechanisms that control their transcription resulting in HLA class I molecule down-regulation.

Aberrations in expression of HLA class I derived peptide complex have frequently been observed in several types of cancer both in vivo and in vitro. Their frequency ranges from 0–90%. Depending on tumor types, these defects have been associated with aggressive histopathological features as well as poor survival [133,134,135,136,137,138,139,140,141,142,143,144,145,146,147]. Most of the defects are caused by genetic or epigenetic mutations as well as by transcriptional or post-translational modifications [145,148,149]. These types of alterations can induce a total loss or down-regulation of HLA class I derived peptide complex as well as selective loss of HLA class I haplotypes or alleles (Figure 1) [150,151,152].

A complete loss of HLA class I derived peptide complex requires two genetic events: a mutation in one copy of the wild-type β2-m and loss of the other non-mutated copy. This phenomenon leads to the loss of heterozygosity (LOH), a genetic abnormality frequently found in malignant cells [153,154,155]. The mutations in β2-m can range from large deletions to single nucleotide mutations. Both types of alterations in most cases inhibit the translation of β2-m mRNA or abolish the disulfide linkage required for the native structure of β2-m, preventing its binding to HLA class I heavy chains [138,139,153]. Although mutations in β2-m can be randomly distributed, a mutation hot spot located in the CT repeat region of exon 1 has been identified in more than 75% of tumor cells, reflecting an increased genetic instability of this region during malignant transformation. As a result tumor cells present total HLA class I molecule loss since the HLA class I heavy chain-β2-m-peptide complex is not formed and not transported to the cell membrane [156,157,158,159]. In contrast, selective HLA class I allospecificity loss requires only one genetic event. This involves mutations of HLA class I allele(s) which inhibit HLA class I molecule transcription or translation. The other allele remains intact and no LOH is required. Loss of one HLA class I haplotype, e.g., HLA-A24, -B56, -Cw7, appears to be frequently caused by loss of segments of the short arm of chromosome 6, where HLA class I genes reside. LOH at chromosome 6 represents a frequent mechanism that contributes to selective HLA haplotype loss in tumors [160,161].

In addition, multiple types of alterations can induce down-regulation of HLA class I molecules [150,151,152]. Specifically, transcriptional activity of HLA class I heavy chain genes can be suppressed by: (i) the presence of silencer localized at the distal promoter region of HLA class I heavy chain gene [162,163]; (ii) epigenetic mechanisms which alter chromatin structure of the HLA class I heavy chain gene promoters; and (iii) DNA hypermethylation [162,164,165,166]. It is well known that the constitutive patterns of DNA methylation in solid and hematopoietic human malignancies are characterized by global hypomethylation with concomitant localized hypermethylation of DNA [148,167,168]. Furthermore, an impaired function of one of the APM components can also reduce the expression of HLA class I derived peptide complex [145]. Lastly, down-regulation of HLA class I antigen complex can be caused by alterations in the transcription factors forming the enhanceosome which bind SXY module on HLA class I heavy chain promoters [148,169,170,171,172]. Specifically, the expression and function of the NLRC5/CITA trans-activator can be affected by promoter methylation, copy number loss and somatic mutations [173,174]. About 60% of somatic mutations result in the inactivation of NLRC5/CITA [174]. 

### 2.2. Proteasome Defects

Alterations of proteasome subunits have been identified by utilizing mAbs that allow semi-quantitative analyses of the constitutive subunits δ, Z and MB1, as well as of the immunoproteasome subunits LMP2, LMP7 and LMP10 [25]. Down-regulation of one of these proteins caused by mutations at coding microsatellites or single nucleotide polymorphisms have been described in several types of tumors including colorectal, bladder, and ovarian carcinomas, as well as in acute myeloid leukemia and melanoma [147,175,176,177,178,179]. As previously described, the proteasome plays a key role in immune regulation. Consequently, inhibition or loss of function in one of the proteasomal components inhibits antigen processing and presentation and modifies the characteristics of processed peptides, decreasing the efficiency of epitope generation and altering tumor cell recognition by naïve T cells [25,169,171].

### 2.3. Defects in TAP1, TAP2 and Other Chaperones

Among the APM components, TAP genes have been most extensively investigated. At genetic level, mutations in TAP genes, resulting in total protein loss or expression of a non-functional protein, have been described in breast, lung, gastric, colorectal, and cervical carcinomas. Their frequency ranges from 10-84% in the cases analyzed [100,180,181,182,183,184]. TAP abnormalities reduce the translocation of peptides into the ER, resulting in a decreased formation of stable HLA class I derived peptide complexes or expression of “peptide-free” HLA class I molecules [171,172]. Interestingly, TAP-deficient individuals do not succumb to viral infections, suggesting that CD8+ T-cell immunity is sufficiently supported by an increased number of alternative TAP-independent processing pathways [171,172]. Identification of these alternative loading mechanisms into peptide-receptive HLA class I molecules still needs further investigation. It is reported that peptides can walk on multiple different paths before ending up in the grooves of HLA class I molecules [185]. Lastly, a substantial down-regulation in chaperone expression have been also associated to several types of malignancies due to defects in proper loading and assembly of HLA class I molecules, altering their maturation and stability [25,88].

### 2.4. HLA Class II Defects

Contrasting results have been described about the clinical significance of alterations in HLA class II molecule expression in cancer. Defects in HLA class II pathway, as well as induction of HLA class II molecule expression by non-immune cells have been involved in carcinogenesis [100]. In addition, HLA class II expression by cancer cells has been associated with poor prognosis and disease progression in melanoma and osteosarcomas [186,187,188]. However, an improved overall survival has been also associated to HLA class II expression by cancer cells in several types of cancer including melanoma, laryngeal, breast, cervical and colorectal cancer [100,188,189,190,191,192,193,194,195,196,197]. In various type of cancer (plasmacytoma, small cell lung cancer, and hepatocarcinoma) defects in HLA class II molecules have been associated to CIITA defects. The latter results in a reversible detrimental HLA class II expression that can be restored by CIITA transfection [100,198,199,200]. Moreover, other HLA class II presentation antigen pathway defects have been also described in Hodgkin’s disease cancer cells [100,201].

## 3. Role of HLA as A Predictive Biomarker for ICI-Based Immunotherapy

### 3.1. Impact of HLA Class I and II on ICI-Based Immunotherapy In Vivo

As we have described above, HLA class I antigen derived peptide complex is crucial for tumor antigen presentation to naïve T cells. Binding of HLA class I antigen derived peptide complex to the TCR of naïve T cells allows T cell activation and consequently the recognition and the lysis of altered tumor cells [7,15,16,17,18]. However, binding of HLA class I derived peptide complex to TCR is not sufficient to activate naïve T cells. Naïve T cell activation requires the interaction between the CD28 family receptors on T cell with their co-stimulatory ligands belonging to B7 family molecules on APCs. Therefore, T cell activation is tightly controlled by co-stimulating or co-inhibiting signaling which are triggered by the interaction between immune checkpoint molecules such as PD-1 and CTLA-4 and their ligands PD-L1 and CD80/CD86 [202,203,204,205], expressed on naïve T cells and APCs, respectively. Actually, the interaction between PD-1 and PD-L1 is crucial in the activation phase of T cells as well as in their effector phase, due to PD-L1 expression also on tumor cells. Several lines of evidence, both in vitro and in vivo, have shown that blockade of the co-inhibitory signaling, including PD-1/PD-L1 axis, by mAbs promotes a host immune response against cancer cells by releasing T cells activation [206]. This novel therapeutic approach, called ICI-based immunotherapy, is revolutionizing the treatment of solid tumors [207]. Several clinical trials in various types of malignancies, such as melanoma, head and neck, triple negative breast, lung, kidney and bladder cancer, have demonstrated that administration of mAbs, which inhibit the interaction of immunoregulatory checkpoint molecules, such as CTLA-4 and PD-1, with their ligands CD80, CD86, and PD-L1, can have a major and lasting effect on their clinical course, significantly improving clinical outcomes as compared with standard chemotherapy [208]. However, this type of therapy is effective only in a subgroup of cancer patients, regardless of the tumor type. Therefore, there is an urgent need to identify the mechanisms of resistance as well as predictive biomarkers which may help to select patients who may benefits from this type of therapy [209,210,211]. Several molecules have been investigated as potential predictive biomarkers of ICI-based immunotherapy [212,213,214,215]. Among the postulated escape mechanisms utilized by tumor cells to avoid recognition and destruction by the host’s immune system, are defects in the ability of tumor cells to process and present tumor antigens to naïve T cells [216]. This phenomenon is mediated by defects in the expression of HLA class I antigen-tumor antigen derived peptide complexes. Therefore, there has been an interest in investigating whether decreased or complete loss of HLA class I and II molecules as well as defects in the APM molecules might predict the efficacy of ICI-based immunotherapy by impairing naïve T cells activation induced by anti-checkpoint molecules. Several lines of evidence in vivo indicate that HLA class I or II modulation play a major role in the efficacy of ICI-based immunotherapy and various humanized mouse models that reliably reflect the complexity of the human heterogeneous tumour and its TME, have been developed in order to evaluate the potential role of HLA class I and II in predicting the efficacy of ICI-based immunotherapy as well as immune adverse effects for different types of cancer [217]. In the study of Lechner MG et al., six murine solid tumor models (CT26, 4T1, MAD109, RENCA, LLC, and B16) were used to demonstrate that MHC class I expression on tumor cells is an excellent surrogate marker of the overall tumor immunogenicity level as well as a predictor of response to immunotherapy. Specifically, tumor growth rate correlated indirectly with MHC class I expression and overall immunogenicity of the tumor model, with fastest growth in B16, LLC, and MAD109 and slowest growth in CT26, RENCA, and 4T1 [218]. Ashizawa et al. reported that HLA class I and class II KO NOG mice (NOG dKO) transplanted with human PBMCs and tumor cell lines showed high anticancer effects following a PD-1 antibody treatment [219]. Gettinger et al. functionally demonstrated that loss of HLA class I expression by CRISPR-mediated knock-out of β2-m in an immunocompetent cancer mouse model (A/J mice transplanted with murine lung cancer cell line UN-SCC680AJ) confers resistance to PD-1 blockade and tumour progression [220]. β2-m gene deactivation in a mouse oncogenic TC-1 cell line derived from primary lung epithelial cells has also been shown to lead to negative surface MHC-I expression along with reduced proliferation and tumor rejection. Despite stimulation with IFN-γ, tumour cells were only weakly responsive to combined immunotherapy [221]. In addition to HLA class I expression, it is important to acknowledge that HLA haplotypes have also been shown to correlate with immunotherapy response in vivo. Rangan L et al. described a tumor cell line generated from a naturally occurring tumor in HLA-A*0201/DRB1*0101 (A2/DR1) mouse named SARC-L1 with a very low expression of HLA-A*0201 molecules, absence of HLA-DRB1*0101 and weak but constitutive expression of PD-L1. Histological and genes signature analysis supported the sarcoma origin of this cell line. According to the high frequency of these HLA alleles in the world population, this mouse model gained considerable interest in the field of tumor immunology and it has been used as preclinical tool for the evaluation of antitumor immunotherapies. Interestingly, both HLA-A*0201 and PD-L1 expressions increased on SARC-L1 after IFN-γ exposure in vitro defining this tumor very sensitive to several drugs commonly used to treat sarcoma and susceptible to anti-PD-L1 mAb therapy in vivo [222]. As we have described above, tumor cells might also express tsHLA-II molecules [123,124,125,126,127,128,129,130]. In the majority of studies, tsHLA-II molecule expression by cancer cells is associated with better prognosis, improved response to ICI in humans and increased tumor rejection in mouse models of breast cancer, sarcoma, lung cancer and colon cancer [98]. However, contrasting reports have shown that tsHLA-II or CIITA has no effect or, in some cases, accelerates tumor growth. In a mouse model of lung cancer Mortara L et al. demonstrated that single cell clones derived from a CIITA transduced population grew more aggressively in mice when cell surface MHC-II was highly expressed [223]. These contrasting results are likely to reflect different variables present in the different mouse model and cancer cells utilized, including (i) the ability of TAAs to be presented differentially on MHC class I or II in each model system, (ii) the number of mutations and therefore number of candidate neo-antigens expressed, (iii) the number of tumor cells injected, (iv) the injection site of cancer cells in the mouse, and (v) the mouse strains used that are characterized by different immunological status. An intriguing but underexplored hypothesis is that induction of HLA class I-II molecules may lead to up-regulate immunoinhibitory molecules on tumor-infiltrating lymphocytes (TILs), such as lymphocyte activation gene 3 (LAG-3) that binds HLA class II and negatively regulates cellular proliferation, activation and homeostasis of T cells, in a similar fashion to CTLA-4 and PD-1. This phenomenon has been reported to play a role in regulatory T cell (Treg) suppressive function creating a tolerizing microenvironment for tumor growth. ICIs directed to LAG-3 have been shown to have synergy with PD-1 inhibition in mouse models, suggesting that co-signaling blockade could restore a favorable immune microenvironment that can respond to antigenic stimulation [224].

### 3.2. HLA Class I and II as Predictive Biomarker for ICI-Based Immunotherapy: Clinical Evidences

Actually, few clinical studies have been investigating the potential role of HLA class I and II antigens in predicting the efficacy of ICI-based immunotherapy and no large clinical cohort analysis of patient population has been performed. Rodig et al. retrospectively evaluated whether HLA proteins confer differential sensitivity to CTLA-4 and PD-1 blockade in pre-treated metastatic melanoma patients. Tumor biopsies were obtained from patients enrolled in two different trials: CheckMate 064 and CheckMate 069. In these trials, patients were treated with the anti-CTLA-4 monoclonal antibody ipilimumab followed by the anti-PD-1 mAb nivolumab, nivolumab followed by ipilimumab, ipilimumab alone, or concurrent nivolumab plus ipilimumab. In this study, Rodig et al. demonstrated that (i) reduced tumor HLA class I molecule expression (≤ 30%) correlated with lack of response to ipilimumab; (ii) HLA class II molecule expression (>1%) correlated with tumor response to nivolumab; and (iii) among nivolumab plus ipilimumab treated patients, reduced HLA class I molecule expression was not associated with progressive disease as well as a decreased tumor response and overall survival. As a result, HLA class I molecule expression appeared a reliable predictive biomarker of tumor response to anti-CTLA-4 mAb ipilimumab but not to anti-PD-1 mAb nivolumab. In contrast, HLA class II molecule expression might represent a useful predictive biomarker for nivolumab but not ipilimumab therapy [126]. In addition, Chowell et al. retrospectively performed high-resolution HLA class I genotyping of 1535 advanced cancer patients treated with ICI-based immunotherapy. Patients affected by non-small cell lung cancer or melanoma were treated with anti-CTLA-4, anti-PD-1/PD-L1 or combinations of both. Results from this study demonstrated that patients carrying maximal heterozygosity at HLA class I loci (HLA-A, HLA-B, or HLA-C) have an improved overall survival as compared to patients who were homozygous for at least one HLA locus. Moreover, patients carrying HLA-B44 supertype had extended survival, while those carrying HLA-B62 supertype (including HLA-B*15:01) or somatic loss of heterozygosity at HLA class I had poor survival outcomes [225].

## 4. Restoring HLA Class I Expression as A Novel Therapeutic Strategy for Cancer Immunotherapy

Identification of the molecular aberrations responsible for altered tumor expression of HLA class I derived peptide complexes is crucial for the success of cancer T cell-based immunotherapy as well as for the rational design of novel immunotherapeutic strategies which restore an integral expression of HLA class I derived peptide complexes. Most of the defects of HLA class I derived peptide complex in human cancers are distinguished in “hard” or “soft” lesions [152]. “Hard” lesions are caused by structural gene alterations that induce loss of expression of HLA class I derived peptide complexes. They are reported in about 30–40% of human cancers [226]. LOH and β2-m gene mutations at chromosomes 6 and 15, respectively, represent the major cause of “hard” defects. In addition, a homologous point mutation in codon 67 of the β2-m gene also results in the total loss of HLA class I molecule expression [227,228]. These types of alterations cannot be repaired by any signaling pathway inhibitors as well as chemotherapeutic or immunotherapeutic agents. Del Campo et al. showed that infection of cells carrying β2-m mutations with an adenoviral vector expressing the human β2-m gene caused a total restoration of HLA class I molecule expression [229]. Thus, based on the type of mutated genes, transfection with a wild type gene, such as HLA class I heavy chain or β2-m genes, can potentially restore the expression of HLA class I derived peptide complex [152].

**Figure 2:**
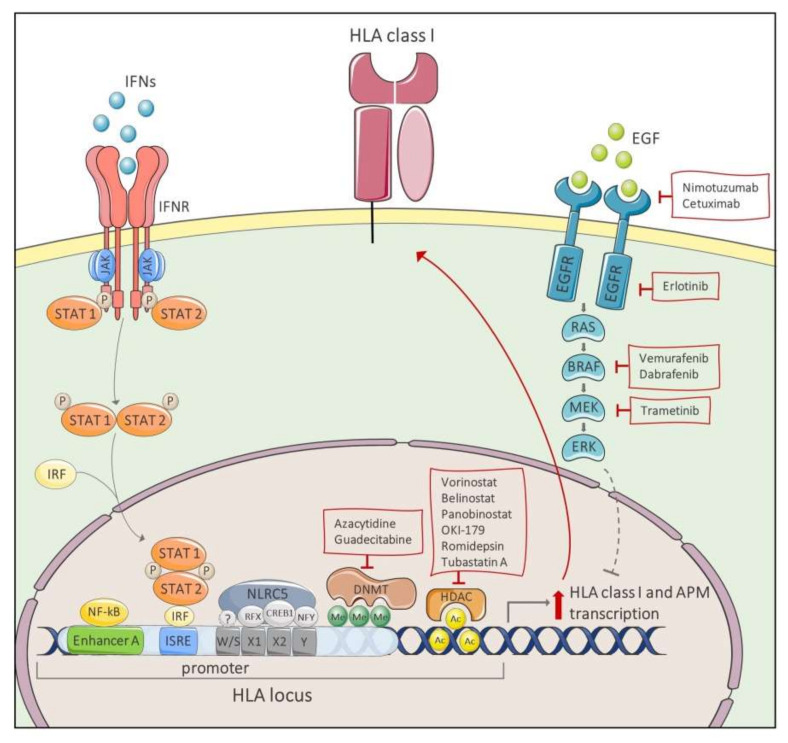
Mechanisms for restoring HLA class I expression. IFN binding to IFNR triggers Jak/STAT transduction pathway. STAT1/STAT2/IRF complex translocates to the nucleus where it binds to ISRE motifs located in HLA promoter region, inducing HLA gene transcription. EGFR and MAPK down-stream pathways suppress HLA class I surface expression. EGFR inhibitors, such as nimotuzumab, cetuximab and erlotinib, BRAF inhibitors, vemurafenib and dabrafenib, and MEK inhibitor trametinib can increase expression of both HLA class I antigens and APM components. DNMT inhibitors (azacytidine and guadecitabine) and HDAC inhibitors (vorinostat, belinostat, panobinostat, OKI-179, romidepsin and tubastatin A) avoid hypermethylation of HLA promoter region and histones hypoacetylation that cause HLA genes silencing. Abbreviations: HLA, human leukocyte antigen; IFNs, interferons; IFNR, interferon receptor; IRF, IFN regulatory factor; ISRE, IFN-sensitive response element; EGF, epidermal growth factor; EGFR, epidermal growth factor receptor; DNMT, DNA methyltransferase; HDAC, histone deacetylase; APM, antigen-processing machinery.

On the other hand, “soft” defects are caused by transcriptional or post-transcriptional modifications of one of HLA class I APM component genes. Activation of pro-tumorigenic pathways or epigenetic modifications which induce a reduced expression of HLA class I APM components are the major causes of “soft” defects [230]. Activation of pro-tumorigenic pathways includes inhibition of Jak/STAT pathway or activation of mitogen-activated protein kinase (MAPK) pathway. Epigenetic modifications include those that cause impairment in gene regulation such as hypermethylation of the HLA-A, B, and C heavy chains, β2m and APM component encoding gene promoter regions, or unbalanced histone acetylation [152,227,228,231]. Lastly, post-transcriptional alterations are induced by aberrant function of micro-RNAs (miRNAs). All these types of alterations can be restored by signaling pathway inhibitors, chemotherapeutic or immunotherapeutic agents (Figure 2). In Table 1, we have summarized the information available in the literature on the main molecules able to restore HLA class I expression in cases associated with “soft” defects.

The administration of IFNs might effectively counteract HLA class I down-regulation in cancer cells by boosting the presentation of tumor specific-associated antigens [254,255,256,257]. A phase 0 clinical trial showed that systemic administration of IFN-γ increases not only HLA class I expression on tumor cells, but also T-cell infiltration in cold tumors [258] IFN-mediated up-regulation of HLA class I derived peptide complexes occurs via activation of the Jak/STAT pathway which induces the binding of IRFs to ISRE motifs located in HLA class I promoter region [107,254]. In addition, IFN-γ-mediated HLA class I derived peptide complex up-regulation is also associated to an increased histone demethylation and acetylation of APM genes in the MHC locus, particularly of histone H3 in TAP1 promoter locus [259]. Down-regulation of Jak/STAT signal transduction pathway and therefore of HLA class I derived peptide complex is strictly linked to protein kinase activation. Activation of either epithelial growth factor receptor (EGFR) or Ras/MAPK downstream pathway directly suppresses expression of HLA class I derived peptide complex and IFN-induced antigen presentation [232,233]. In this case the combination of IFNs and tyrosine kinase inhibitors (TKIs) can represent a potential therapeutic strategy for recovering HLA class I derived peptide complex expression in cancer cells. EGFR inhibitors such as the monoclonal antibodies nimotuzumab and cetuximab and the tyrosine kinase inhibitor erlotinib have been shown to increase membrane expression of HLA class I APM components in cells with EGFR activation [234,235,236]. Im and collaborators showed that the sensitivity of lung cancer cells to erlotinib positively correlates with the increase of expression of HLA class I derived peptide complex following IFN-γ treatment [235]. Moreover, the MAPK/ERK kinase (MEK) inhibitor trametinib, in combination with IFN-γ, has been shown to enhance tumor immunogenicity by modulating HLA class I derived peptide complex in different types of malignancies [232], including triple-negative breast cancer [233] and head and neck squamous cell carcinoma [237]. In melanoma patients harboring the BRAF^V600E^ mutations, BRAF inhibitor vemurafenib strengthens the induction of HLA class I antigen expression by both IFN-γ and IFN-α2b [238,239]. Furthermore, this effect was enhanced when BRAF inhibition was combined with the MEK inhibitor trametinib [240].

However, it is important to mention that several tumors develop IFN-γ signaling insensitivity. The lack of response to IFN-γ stimulation results from cellular defects on IFNγR1receptor or downstream components of the signaling pathway such as Janus kinases (JAK) 1 and 2 [260]. In addition, the absence of STAT1 expression and/or tyrosine-phosphorylation and epigenetic regulation of IRF1 can contribute to the lack of HLA class I expression, restoring in vitro following IFN-γ administration [261,262]. Lastly, given the addiction of IFN-γ-induced HLA class I and II up-regulation by NLRC5/CITA and CIITA, respectively, genetic or functional defects on these two trans-activators abrogate the ability of IFN-γ to boost antigen processing and presentation [106,260]. 

Inhibition of histone deacetylases (HDACs), enzymes that remove acetyl group from lysines on histones, also promotes the increase of expression of HLA class I APM components. HDAC inhibitors (HDACi) vorinostat, belinostat, and panobinostat, targeting HDAC class I, II, and IV, and romidepsin, a specific HDAC 1 and 2 inhibitor, are currently approved for hematological malignancies [228,230]. Vorinostat, also known as SAHA, in combination with mithramycin A, a Sp1 inhibitor, reverses the histone hypoacetylation which causes APM gene silencing in Merkel cell carcinoma [241]. In addition, vorinostat promotes tumor cell recognition by CTLs in glioma cells [242,243]. In diffuse large B-cell lymphoma, the HDAC class I inhibitor OKI-179 reverts down-regulation of HLA class I derived peptide complex, a typical feature of this hematological cancer [230]. Likewise, the selective inhibition of HDAC6 with tubastatin A improves the immunogenicity of melanoma cells by increasing HLA class I expression [244]. Mutations in EZH2 gene, encoding for a histone-lysine N-methyltransferase, are strictly connected to loss of expression of HLA class I derived peptide complex in large B-cell lymphoma. Treatment with EZH2 inhibitor tazemetostat (EPZ-6438) restores the expression of HLA class I derived peptide complex in EZH2-mutant cell lines [245]. In cases of DNA hypermethylation, therapy with DNA methyltransferase inhibitors (DNMTi) such as azacytidine and decitabine restores the expression of HLA class I derived peptide complex [263]. Azacytidine increased the transcription of APM genes and HLA class I molecule expression in lung carcinoma and melanoma cells [246,247]. Guadecitabine, a novel DNMTi, significantly up-regulated both basal and IFN-γ-dependent expression of HLA class I derived peptide complex in breast cancer cells [248]. In the case of HLA class I APM component down-regulation mediated by aberrant miRNA function, it has been shown that suppression of miR-9 and miR-19 expression restores the expression of HLA class I derived peptide complex [264,265]. Lastly, several chemotherapeutic agents can promote the expression of HLA class I derived peptide complex. In many types of cancer cell lines cisplatin alone or in combination with vinorelbine or 5-fluorouracil [249], doxorubicin [250], the microtubule-destabilizers epothilone B, taxol and vinblastine [251] increase the expression of HLA class I APM components. Moreover, some other chemotherapeutic agents, such as the topoisomerase-I inhibitors topotecan and etoposide, indirectly induce the up-regulation of HLA class I derived peptide complex by stimulating IFN-β autocrine/paracrine signaling of tumor cells [252].

## 5. Conclusions

In the last decades, important progress has been made pertaining to the knowledge of the structure and the function of HLA class I and II antigen presentation pathways. An improved knowledge of molecular mechanisms underlying HLA class I APM defects will be crucial to better understand the mechanisms determining carcinogenesis, tumor progression and immune escape. Moreover, recent knowledge of the pathways leading to restored HLA expression could be utilized to conceive new cancer therapeutic strategies. The identification of molecular defects resulting in acquired insensitivity to the stimulation with specific adjuvants, such as IFNs, could allow to a better selection of cancer patients who can take advantage from this approach for HLA expression restoring. Because of their crucial role in tumor cell recognition, additional studies are urgently needed to validate the role of expression of HLA class I and II antigens, as well as of APM components as novel potential predictive biomarkers in cancer immunotherapy. Moreover, where HLA class I and II gene expression is not recoverable, the development of different immunotherapeutic approaches able to target native cell surface antigens, and therefore independent from HLA expression, such as chimer antigen receptor (CAR)-T cell or bi-specific T-cell engager antibodies (BiTES) should be taken in consideration.

## Figures and Tables

**Table 1:** Drugs for restoring MHC class I expression [228,230,232,233,234,235,236,237,238,239,240,241,242,243,244,245,246,247,248,249,250,251,252,253].

Name	Target	Combination Therapy	Cancer Type	References
**Kinase Inhibitors**				
Nimotuzumab Cetuximab Erlotinib	EGFR	IFN-γ	epidermoid carcinoma lung cancer head and neck carcinoma lung adenocarcinoma	Garrido G. et al. 2017 [236] Srivastava et al. 2015 [234] Im et al. 2016 [235]
Trametinib	MEK1/2	IFN-γ	mesothelioma melanoma lung adenocarcinoma colon adenocarcinoma thyroid carcinoma breast cancer head and neck carcinoma	Brea et al. 2016 [232] Loi et al. 2016 [233] Kang et al. 2019 [237]
Vemurafenib	BRAF^V600E^	IFN-γ IFNα-2b	Melanoma	Sapkota et al. 2013 [238] Sabbatino et al. 2016 [239]
Dabrafenib		Trametinib		Hu-Lieskovan et al. 2015 [240]
**Epigenetic Agents**				
Vorinostat	HDAC class I, II, and IV	Mithramycin A	Merkel cell carcinoma	Ritter et al. 2017 [241]
			B-cell lymphoma cutaneous T-cell lymphoma acute myeloid leukemia glioma	Chacon et al. 2016 [247] Banik et al. 2019 [228] Sun et al. 2019 [242] Yang et al. 2019 [243] Wang et al. 2020 [253]
Belinostat			peripheral T-cell lymphoma B-cell lymphoma	Banik et al. 2019 [228] Wang et al. 2020 [253]
Panobinostat			multiple myeloma B-cell lymphoma	Banik et al. 2019 [228] Wang et al. 2020 [253]
OKI-179			diffuse large B-cell lymphoma	Wang et al. 2019 [230]
Romidepsin	HDAC 1-2		B-cell lymphoma peripheral T cell lymphoma	Banik et al. 2019 [228] Wang et al. 2020 [253]
Tubastatin A	HDAC 6		Melanoma	Woan et al. 2015 [244]
Azacytidine	DNMT		lung carcinoma melanoma	Fonsatti et al. 2007 [246] Chacon et al. 2016 [247]
Guadecitabine			breast cancer	Luo et al. 2018 [248]
Tazemetostat	EZH2		diffuse large B-cell lymphoma	Ennishi et al. 2019 [245]
**Chemotherapeutics**				
Cisplatin	DNA	Vinorelbine or5-fluorouracil	lung cancer head and neck carcinoma	De Biasi et al. 2014 [249]
Epothilone B Taxol Vinblastine	Microtubules		ovarian cancer	Pellicciotta et al. 2011 [251]
Doxorubicin	Topoisomerase		nasopharyngeal carcinoma	Faè et al. 2016 [250]
Topotecan Etoposide			breast cancer	Wan et al. 2012 [252]

## Abbreviations

ACT	adoptive cell-transfer
APC	antigen presenting cell
APM	antigen processing machinery
ATF1	activating transcriptor factor 1
ATP	adenosine triphosphate
BiTES	bi-specific T-cell engager antibodies
BLS	bare lymphocyte disease
CAR	chimeric antigen receptor
CIITA	class II trans-activator
CIITA	class I trans activator
CLIP	class II-associated I chain peptide
CNX	calnexin
CREB1	cAMP-responsive element binding protein 1
CRT	calreticulin
CTL	cytotoxic T lymphocyte
CTLA-4	cytotoxic T-lymphocyte antigen 4
DC	dendritic cell
DFS	disease free survival
DNMTi	DNA methyltransferase inhibitor
EGFR	epithelial growth factor receptor
ER	endoplasmic reticulum
HDAC	histone deacetylase
HLA	human leukocyte antigen
ICI	immune checkpoint inhibitor
IFN	interferon
IRF	IFN Regulatory Factor
ISRE	IFN-sensitive response element
KIR	killer cell immunoglobulin-like receptor
JAK	janus kinase
LAG-3	lymphocyte activation gene 3
LMP	low molecular weight protein
LOH	loss of heterozygosity
mAb	monoclonal antibody
MAPK	mitogen-activated protein kinase
MEK	MAPK/ERK kinase
MHC	major histocompatibility complex
MIIC	MHC class II compartment
NF-kB	nuclear factor kB
NFY	nuclear transcriptor factor Y
NK	natural killer
NLRC5	NOD-like receptor 5
OS	overall survival
PD-1	programmed cell death 1
PD-L1	programmed cell death ligand 1
PFS	progression free survival
RFX	regulatory factor X
RFXANK	RFX-associated ankyrin-containing protein
RFXAP	RFX associated protein
TAP	transporters associated with antigen processing
TCR	T-cell receptor
TKI	tyrosine kinase inhibitor
TNF-α	tumor necrosis factor alpha
TA	tumor antigen
TAA	tumor associated antigen
TAM	Type II tumour-associated macrophages
TIL	tumor-infiltrating lymphocyte
TME	tumor microenvironment
Treg	regulatory T cell
TSA	tumor specific antigen
tsMHC-II	tumor specific MHC class II
β2-m	β2-microglobulin
